# Death Certificates and Insurance Companies

**Published:** 1915-08-14

**Authors:** 


### DEATH CERTIFICATES AND INSURANCE COMPANIES.

### The Companies' Attitude and the Doctor's Rights.

After the war one of the subjects likely to engage the attention of the public and the Legislature will be the certification of death. The present position in this matter is admittedly unsatisfactory, and certain formal inquiries have already been instituted in regard both to it and to an allied topic—namely, the Coroner's inquest. When the time comes the medical profession will have much to say on both these matters, and this in relation alike to professional obligations and to the public interest. In the meantime, there are one or two points which deal with procedure and which are capable of being better regulated than the usual custom dictates.

The extent of the obligation incumbent on a medical practitioner in relation to the certificate of death is to record the fact together with the name of the disease which has led to the fatal issue. For the latter of these he gives his personal guarantee, but he may record the actual occurrence of death on the strength of a statement made to him by a relative or other person in attendance on the deceased. In rare instances a doctor has been deceived. Hence it is well when any chance of doubt exists that the practitioner should himself verify the fact of death, though there is no legal obligation placed upon him to do this, and it is absurd to suggest, as has been done by a modern critic, that there is a moral claim in excess of the legal demand. Already the law compels the practitioner to give a certificate without payment or reward, and to add to this gratuitous duty an obligation which would often mean a long journey is really asking a little too much. When the doctor can give his personal testimony to the fact of death let him by all means do so, but when he is depending in this respect upon a statement made by some other person he ought to avail himself of the official opportunity to certify the event as one reported to him, though he himself has not verified it.

In addition to a notification of the fact of death and the cause of it the official certificate provides spaces in which there may be entered the length of time during which the fatal illness is believed to have lasted. To fill up these spaces is not compulsory, and there are circumstances in which it is advisable they should be left blank; indeed, we would put the position more strongly and would say that the circumstances are exceptional in which any entry should be made in them. The first reason for this counsel is that in a great many chronic illnesses any estimate of the duration of the disease is a highly speculative undertaking. Who, for example, can undertake to indicate with any degree of precision the beginnings of malignant disease, or of cirrhosis of the liver, or of granular kidney, or of tabes dorsalis, or of chronic endocarditis? Notoriously such a thing is impossible. Why, then, make a parade of precision?

A second reason for abstaining from filling in any time estimate of the duration of the disease is that such estimate may be used, and we think unfairly used, by certain insurance companies in order to relieve themselves from the responsibility of paying a claim arising under a policy granted on the life of the deceased. What happens is this: An insurance company, by means of agents who call from door to door, persuades numbers of the poorer classes to insure the lives of themselves or their children and collects small weekly payments by way of premium. A form has to be filled up in which the insured person is stated to be in good health, and this is the basis of the contract. Of course, the agent is anxious to do business, and quite possibly the other party to the transaction hardly realises the severe conditions which attend the bargain. All goes well until death occurs, when the medical man who fills up the death certificate ventures upon a rough time estimate of some chronic disease which he has found to be present. Should this estimate involve a date prior to the date on which the insurance was effected the insurance company is very likely to repudiate the policy. The matter is one of business and the company has a plausible case. But this case is made for them gratuitously by a medical practitioner. They choose to effect insurances without the check of medical examination, and, of course, can conduct their business as they think best. But having elected to take this method is it not reasonable that they shall continue without medical assistance to the end of the chapter? As it is, there is only too often reason to fear that the medical profession pulls the chestnuts out of the fire for them. Of course, we are not advocating deceit or pretending to defend it from its proper penalties. But we are saying that when a judgment in reference to the duration of a disease is more or less speculative, it is just as well to avoid entering in the death certificate particulars which the law does not demand, and which are sometimes used, as aids to the successful prosecution of the enterprises of life insurance companies.

The same question in essence arises when, on the death of one of their clients, an insurance company demands a certificate from the practitioner in attendance during the last illness of the deceased. Some of these certificates assume formidable proportions, and though the company wants the information, it does not proposes to pay a fee, as, by a clause in the original contract, the onus of providing the certificate is thrown on the estate of the insured person and not on the company. Particulars of the nature of the fatal illness, of the duration of it, and of the cause of it are invited, and for an obvious business purpose. We do not say that the purpose is an illegitimate one; our object is to suggest that there is no need for the medical practitioner to allow himself to be exploited for its promotion. Some of the companies seem to labour under the illusion that the doctor ought to act as an unpaid agent in their interest, ready and willing to keep his eye on their client, and to serve up any information which they think they can utilise to their advantage. It is necessary to disappoint this expectation, and one means of doing it is to decline to commit oneself to positive propositions in relation either to the duration or to the cause of chronic disease except in so far as such propositions are warranted by confident scientific evidence. Because, to take a single instance, hepatic cirrhosis is often or usually due to excess in the use of alcoholic liquors, this does not warrant the confident opinion that such a factor was the responsible agent in any given individual case.

